# *trans*-Diacetonitrile­tetra­kis(1*H*-pyrazole-κ*N* ^2^)nickel(II) dinitrate

**DOI:** 10.1107/S1600536809049472

**Published:** 2009-11-25

**Authors:** Chien-Hong Chen, Chang-Chih Hsieh, Hon Man Lee, Yih-Chern Horng

**Affiliations:** a School of Applied Chemistry, Chung Shan Medical University, Taichung City 40201, Taiwan; b Department of Medical Research, Chung Shan Medical University Hospital, Taichung City, Taiwan; c Department of Chemistry, National Changhua University of Education, Changhua 50058, Taiwan

## Abstract

In the title complex, [Ni(CH_3_CN)_2_(C_3_H_4_N_2_)_4_](NO_3_)_2_, the cation lies on an inversion center and adopts an octa­hedral coordination geometry about the Ni atom. The two acetonitrile ligands are in a *trans* conformation. N—H⋯O hydrogen bonds between cations and anions link the complex mol­ecules into one-dimensional chains running parallel to [100].

## Related literature

For general background and the structures of other salts of this cation, see: Hsieh *et al.* (2009 ▶). 
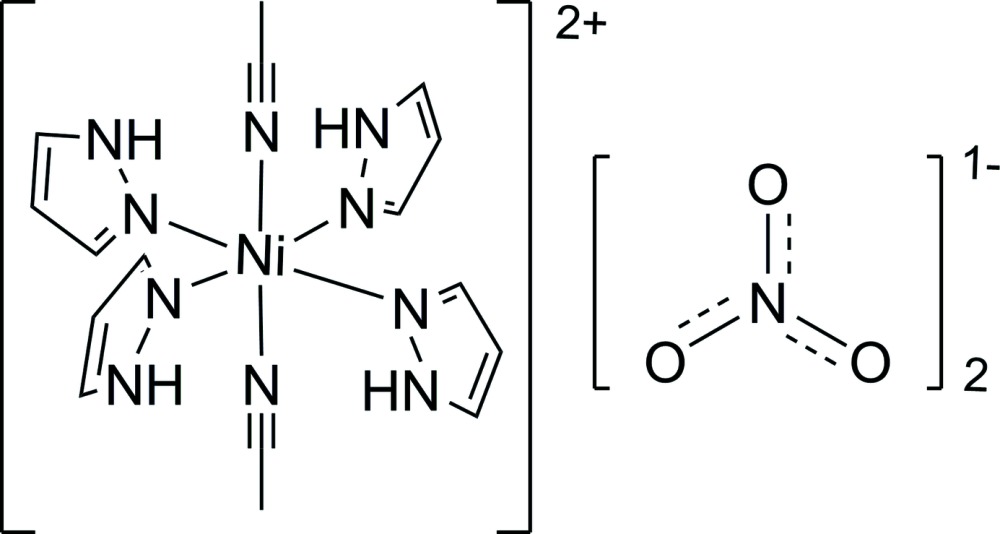


## Experimental

### 

#### Crystal data

[Ni(C_2_H_3_N)_2_(C_3_H_4_N_2_)_4_](NO_3_)_2_
*M* *_r_* = 537.17
Monoclinic, 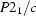

*a* = 9.9815 (5) Å
*b* = 15.2831 (8) Å
*c* = 7.6845 (4) Å
β = 98.817 (2)°
*V* = 1158.40 (10) Å^3^
*Z* = 2
Mo *K*α radiation
μ = 0.90 mm^−1^
*T* = 150 K
0.32 × 0.23 × 0.15 mm

#### Data collection

Bruker APEXII diffractometer
Absorption correction: multi-scan (*SADABS*; Sheldrick, 1996 ▶) *T*_min_ = 0.762, *T*_max_ = 0.877
13134 measured reflections
2992 independent reflections
2247 reflections with *I* > 2σ
*R*_int_ = 0.038

#### Refinement

*R*[*F* ^2^ > 2σ(*F* ^2^)] = 0.047
*wR*(*F* ^2^) = 0.149
*S* = 1.09
2992 reflections
161 parameters
3 restraints
H-atom parameters constrained
Δρ_max_ = 1.13 e Å^−3^
Δρ_min_ = −0.49 e Å^−3^

### 

Data collection: *APEX2* (Bruker, 2007 ▶); cell refinement: *SAINT* (Bruker, 2007 ▶); data reduction: *SAINT*; program(s) used to solve structure: *SHELXSL97* (Sheldrick, 2008 ▶); program(s) used to refine structure: *SHELXTL* (Sheldrick, 2008 ▶); molecular graphics: *SHELXTL*; software used to prepare material for publication: *DIAMOND* (Brandenburg, 1999 ▶).

## Supplementary Material

Crystal structure: contains datablocks I, global. DOI: 10.1107/S1600536809049472/pv2232sup1.cif

Structure factors: contains datablocks I. DOI: 10.1107/S1600536809049472/pv2232Isup2.hkl

Additional supplementary materials:  crystallographic information; 3D view; checkCIF report

## Figures and Tables

**Table 1:** Hydrogen-bond geometry (Å, °)

*D*—H⋯*A*	*D*—H	H⋯*A*	*D*⋯*A*	*D*—H⋯*A*
N4—H8⋯O1^i^	0.88	1.94	2.797 (4)	164
N2—H4⋯O3	0.88	1.95	2.782 (3)	158

Symmetry code: (i) 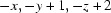.

**Fig. 1.**
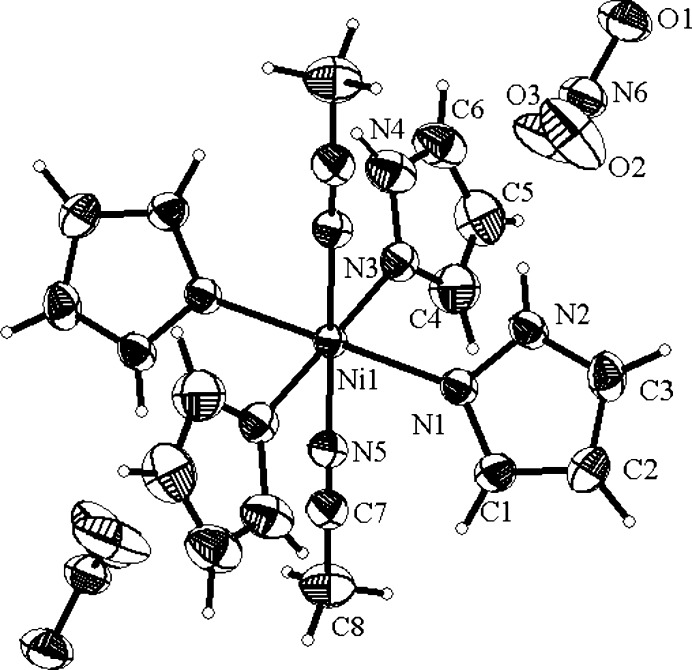
The structure of the title complex, showing 50% displacement ellipsoids; the H atoms are dipicted by circles of an arbitrary radius. Unlabeled atoms of the complex are related to labeled atoms by the symmetry operation: 1 - x, 1 - y, 2 - z.

**Fig. 2.**
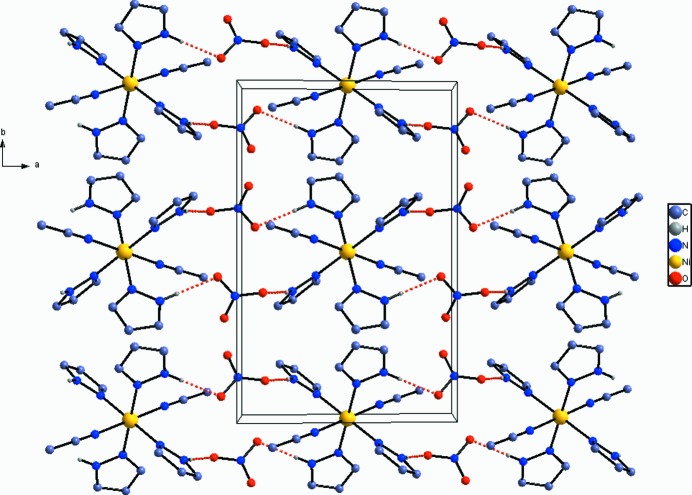
A packing diagram of the title compound along the [001] direction showing the intermolecular hydrogen bonded network (dashed lines).

## supplementary crystallographic information

### Comment

In the title complex (Fig. 1), the Ni atom lies on an inversion center and adopts an octahedral coordination geometry. The two acetonitrile ligands are in a *trans* conformation. The classocal intermolecular hydrogen bonds of the type N—H···O between cations and anions link the complex into one-dimensional chains (Table 1). For general background and the structures of other salts of this cation, see: Hsieh *et al.* (2009).

### Experimental

A solution of Ni(NO_3_)_2 _. 6H_2_O (0.29 g, 0.97 mmol) and pyrazole (0.30 g, 4.30 mmol) in MeCN (25 ml) was stirred at room temperature for 10 min. After the resultant bluesolution was filtered and concentrated to 5 ml under vacuum, the concentrated filtrate was layered with diethyl ether (5-fold portion) and then kept at room temperature for 3 days. The air-stable blue crystals of the title compound (0.39 g, 74%) obtained were suitable for *X*-ray crystallographic analysis.

### Refinement

All the H atoms were positioned geometrically and refined as riding atoms, with C_methine_—H = 0.95, C_methyl_—H = 0.98 and N—H = 0.88 Å while *U*_iso_(H) = 1.2*U*_eq_(C_methine_ and N) and *U*_iso_(H) = 1.5*U*_eq_(C_methyl_). In the final difference map, the highest peak was 1.13 eÅ^-3^ (located in the center of the pyrazole ring N3/N4/C4/C5/C6) and the deepest hole was -0.49 eÅ^-3^ (0.48 Å from N4).

### Crystal data

**Table:**

[Ni(C_2_H_3_N)_2_(C_3_H_4_N_2_)_4_](NO_3_)_2_	*F*(000) = 556
*M**_r_* = 537.17	*D*_x_ = 1.540 Mg m^−^^3^
Monoclinic, *P*2_1_/*c*	Mo *K*α radiation, λ = 0.71073 Å
Hall symbol: -P 2ybc	Cell parameters from 3410 reflections
*a* = 9.9815 (5) Å	θ = 2.7–25.6°
*b* = 15.2831 (8) Å	µ = 0.90 mm^−^^1^
*c* = 7.6845 (4) Å	*T* = 150 K
β = 98.817 (2)°	Block, blue
*V* = 1158.40 (10) Å^3^	0.32 × 0.23 × 0.15 mm
*Z* = 2	

### Data collection

**Table:**

Bruker SMART APEXII diffractometer	2992 independent reflections
Radiation source: fine-focus sealed tube	2247 reflections with *I* > 2σ
graphite	*R*_int_ = 0.038
ω scans	θ_max_ = 28.7°, θ_min_ = 2.1°
Absorption correction: multi-scan (*SADABS*; Sheldrick, 1996)	*h* = −13→13
*T*_min_ = 0.762, *T*_max_ = 0.877	*k* = −17→20
13134 measured reflections	*l* = −10→10

### Refinement

**Table:**

Refinement on *F*^2^	Primary atom site location: structure-invariant direct methods
Least-squares matrix: full	Secondary atom site location: difference Fourier map
*R*[*F*^2^ > 2σ(*F*^2^)] = 0.047	Hydrogen site location: inferred from neighbouring sites
*wR*(*F*^2^) = 0.149	H-atom parameters constrained
*S* = 1.09	*w* = 1/[σ^2^(*F*_o_^2^) + (0.0852*P*)^2^ + 0.4971*P*] where *P* = (*F*_o_^2^ + 2*F*_c_^2^)/3
2992 reflections	(Δ/σ)_max_ < 0.001
161 parameters	Δρ_max_ = 1.13 e Å^−^^3^
3 restraints	Δρ_min_ = −0.49 e Å^−^^3^

### Special details

**Table:**

Experimental. IR (KBr, n_max_/cm^-1^): 3120w (NH), 2283*m* (C≡ N), 2210*m* (C≡ N). Elem. Anal. Calcd (%) for C_16_H_22_N_12_NiO_6_: C 35.78; H 4.13; N 31.29. Found: C 35.32; H 4.01; N 31.03.
Geometry. All e.s.d.'s (except the e.s.d. in the dihedral angle between two l.s. planes) are estimated using the full covariance matrix. The cell e.s.d.'s are taken into account individually in the estimation of e.s.d.'s in distances, angles and torsion angles; correlations between e.s.d.'s in cell parameters are only used when they are defined by crystal symmetry. An approximate (isotropic) treatment of cell e.s.d.'s is used for estimating e.s.d.'s involving l.s. planes.
Refinement. Refinement of *F*^2^ against ALL reflections. The weighted *R*-factor *wR* and goodness of fit *S* are based on *F*^2^, conventional *R*-factors *R* are based on *F*, with *F* set to zero for negative *F*^2^. The threshold expression of *F*^2^ > σ(*F*^2^) is used only for calculating *R*-factors(gt) *etc*. and is not relevant to the choice of reflections for refinement. *R*-factors based on *F*^2^ are statistically about twice as large as those based on *F*, and *R*- factors based on ALL data will be even larger.

### Fractional atomic coordinates and isotropic or equivalent isotropic displacement parameters (Å^2^)

**Table:**

	*x*	*y*	*z*	*U*_iso_*/*U*_eq_	
C1	0.5375 (3)	0.65817 (19)	0.7527 (4)	0.0381 (6)	
H1	0.6327	0.6507	0.7595	0.046*	
C2	0.4624 (3)	0.7228 (2)	0.6544 (4)	0.0459 (7)	
H2	0.4948	0.7667	0.5837	0.055*	
C3	0.3328 (3)	0.70974 (19)	0.6813 (4)	0.0418 (7)	
H3	0.2559	0.7429	0.6316	0.050*	
C4	0.3550 (4)	0.4130 (3)	0.6557 (5)	0.0562 (8)	
H5	0.4194	0.4355	0.5882	0.067*	
C5	0.2476 (4)	0.3580 (3)	0.5885 (5)	0.0596 (9)	
H6	0.2260	0.3373	0.4710	0.072*	
C6	0.1824 (3)	0.3406 (2)	0.7211 (5)	0.0540 (8)	
H7	0.1043	0.3048	0.7182	0.065*	
C7	0.7342 (3)	0.44589 (18)	0.7733 (4)	0.0370 (6)	
C8	0.8414 (3)	0.4248 (2)	0.6709 (5)	0.0521 (8)	
H9	0.8102	0.4377	0.5464	0.078*	
H10	0.8641	0.3626	0.6846	0.078*	
H11	0.9219	0.4600	0.7130	0.078*	
N1	0.4583 (2)	0.60812 (14)	0.8361 (3)	0.0300 (5)	
N2	0.3329 (2)	0.64146 (14)	0.7908 (3)	0.0344 (5)	
H4	0.2601	0.6210	0.8284	0.041*	
N3	0.3559 (2)	0.43008 (14)	0.8258 (3)	0.0321 (5)	
N4	0.2488 (3)	0.38387 (18)	0.8626 (4)	0.0492 (6)	
H8	0.2243	0.3820	0.9678	0.059*	
N5	0.6507 (2)	0.46282 (15)	0.8523 (3)	0.0326 (5)	
N6	0.0036 (2)	0.62767 (19)	0.8693 (4)	0.0478 (6)	
Ni1	0.5000	0.5000	1.0000	0.02738 (16)	
O1	−0.1214 (2)	0.61818 (17)	0.8375 (3)	0.0568 (6)	
O2	0.0532 (3)	0.6921 (2)	0.9519 (5)	0.0830 (9)	
O3	0.0787 (2)	0.57249 (18)	0.8160 (5)	0.0825 (10)	

### Atomic displacement parameters (Å^2^)

**Table:**

	*U*^11^	*U*^22^	*U*^33^	*U*^12^	*U*^13^	*U*^23^
C1	0.0336 (13)	0.0342 (14)	0.0469 (16)	−0.0011 (11)	0.0078 (11)	0.0069 (12)
C2	0.0505 (17)	0.0364 (16)	0.0508 (18)	−0.0010 (13)	0.0080 (14)	0.0127 (13)
C3	0.0444 (15)	0.0296 (14)	0.0482 (16)	0.0067 (12)	−0.0025 (12)	0.0060 (12)
C4	0.0565 (19)	0.065 (2)	0.0475 (15)	−0.0069 (17)	0.0077 (14)	−0.0042 (16)
C5	0.063 (2)	0.059 (2)	0.054 (2)	0.0008 (18)	0.0007 (17)	−0.0118 (17)
C6	0.0427 (17)	0.0406 (18)	0.078 (2)	−0.0024 (13)	0.0063 (16)	−0.0045 (16)
C7	0.0355 (13)	0.0302 (14)	0.0446 (15)	−0.0020 (11)	0.0038 (11)	−0.0021 (11)
C8	0.0481 (17)	0.0486 (19)	0.063 (2)	0.0019 (14)	0.0212 (16)	−0.0106 (16)
N1	0.0263 (10)	0.0244 (10)	0.0388 (12)	0.0008 (8)	0.0031 (8)	0.0021 (9)
N2	0.0280 (10)	0.0261 (11)	0.0472 (13)	0.0001 (8)	−0.0005 (9)	0.0031 (9)
N3	0.0334 (11)	0.0241 (11)	0.0384 (11)	0.0016 (8)	0.0040 (9)	0.0006 (9)
N4	0.0395 (13)	0.0452 (15)	0.0627 (16)	−0.0042 (11)	0.0069 (11)	−0.0050 (13)
N5	0.0299 (10)	0.0270 (11)	0.0414 (12)	0.0005 (9)	0.0072 (9)	−0.0003 (10)
N6	0.0319 (12)	0.0479 (15)	0.0629 (17)	−0.0029 (11)	0.0052 (11)	0.0070 (13)
Ni1	0.0254 (2)	0.0217 (2)	0.0352 (3)	0.00207 (16)	0.00500 (17)	0.00278 (18)
O1	0.0320 (10)	0.0643 (15)	0.0750 (16)	−0.0002 (10)	0.0110 (10)	0.0051 (13)
O2	0.0547 (16)	0.080 (2)	0.104 (2)	0.0142 (15)	−0.0210 (16)	−0.0326 (18)
O3	0.0345 (12)	0.0513 (16)	0.163 (3)	−0.0029 (11)	0.0188 (16)	−0.0222 (17)

### Geometric parameters (Å, °)

**Table:**

C1—N1	1.333 (3)	C8—H9	0.9800
C1—C2	1.391 (4)	C8—H10	0.9800
C1—H1	0.9500	C8—H11	0.9800
C2—C3	1.355 (4)	N1—N2	1.346 (3)
C2—H2	0.9500	N1—Ni1	2.081 (2)
C3—N2	1.340 (4)	N2—H4	0.8800
C3—H3	0.9500	N3—N4	1.347 (3)
C4—N3	1.332 (4)	N3—Ni1	2.100 (2)
C4—C5	1.398 (5)	N4—H8	0.8800
C4—H5	0.9500	N5—Ni1	2.097 (2)
C5—C6	1.318 (5)	N6—O2	1.234 (4)
C5—H6	0.9500	N6—O3	1.238 (4)
C6—N4	1.355 (5)	N6—O1	1.243 (3)
C6—H7	0.9500	Ni1—N1^i^	2.081 (2)
C7—N5	1.134 (3)	Ni1—N5^i^	2.097 (2)
C7—C8	1.458 (4)	Ni1—N3^i^	2.100 (2)
			
N1—C1—C2	111.0 (2)	C3—N2—N1	111.6 (2)
N1—C1—H1	124.5	C3—N2—H4	124.2
C2—C1—H1	124.5	N1—N2—H4	124.2
C3—C2—C1	105.1 (3)	C4—N3—N4	102.5 (3)
C3—C2—H2	127.5	C4—N3—Ni1	128.8 (2)
C1—C2—H2	127.5	N4—N3—Ni1	128.40 (19)
N2—C3—C2	107.6 (2)	N3—N4—C6	113.2 (3)
N2—C3—H3	126.2	N3—N4—H8	123.4
C2—C3—H3	126.2	C6—N4—H8	123.4
N3—C4—C5	111.7 (3)	C7—N5—Ni1	177.3 (2)
N3—C4—H5	124.1	O2—N6—O3	119.9 (3)
C5—C4—H5	124.1	O2—N6—O1	120.4 (3)
C6—C5—C4	106.1 (3)	O3—N6—O1	119.7 (3)
C6—C5—H6	126.9	N1—Ni1—N1^i^	180.000 (1)
C4—C5—H6	126.9	N1—Ni1—N5	88.92 (9)
C5—C6—N4	106.4 (3)	N1^i^—Ni1—N5	91.08 (9)
C5—C6—H7	126.8	N1—Ni1—N5^i^	91.08 (9)
N4—C6—H7	126.8	N1^i^—Ni1—N5^i^	88.92 (9)
N5—C7—C8	179.5 (3)	N5—Ni1—N5^i^	180.00 (12)
C7—C8—H9	109.5	N1—Ni1—N3^i^	92.05 (8)
C7—C8—H10	109.5	N1^i^—Ni1—N3^i^	87.95 (8)
H9—C8—H10	109.5	N5—Ni1—N3^i^	90.28 (9)
C7—C8—H11	109.5	N5^i^—Ni1—N3^i^	89.72 (8)
H9—C8—H11	109.5	N1—Ni1—N3	87.95 (8)
H10—C8—H11	109.5	N1^i^—Ni1—N3	92.05 (8)
C1—N1—N2	104.8 (2)	N5—Ni1—N3	89.72 (8)
C1—N1—Ni1	131.92 (18)	N5^i^—Ni1—N3	90.28 (9)
N2—N1—Ni1	123.30 (16)	N3^i^—Ni1—N3	180.0
			
N1—C1—C2—C3	−0.2 (4)	N2—N1—Ni1—N5	145.4 (2)
C1—C2—C3—N2	0.5 (4)	C1—N1—Ni1—N5^i^	146.4 (3)
N3—C4—C5—C6	−0.5 (5)	N2—N1—Ni1—N5^i^	−34.6 (2)
C4—C5—C6—N4	0.0 (4)	C1—N1—Ni1—N3^i^	56.7 (3)
C2—C1—N1—N2	−0.2 (3)	N2—N1—Ni1—N3^i^	−124.3 (2)
C2—C1—N1—Ni1	178.9 (2)	C1—N1—Ni1—N3	−123.3 (3)
C2—C3—N2—N1	−0.7 (3)	N2—N1—Ni1—N3	55.7 (2)
C1—N1—N2—C3	0.5 (3)	C4—N3—Ni1—N1	60.2 (3)
Ni1—N1—N2—C3	−178.69 (18)	N4—N3—Ni1—N1	−127.2 (2)
C5—C4—N3—N4	0.7 (4)	C4—N3—Ni1—N1^i^	−119.8 (3)
C5—C4—N3—Ni1	174.8 (2)	N4—N3—Ni1—N1^i^	52.8 (2)
C4—N3—N4—C6	−0.7 (3)	C4—N3—Ni1—N5	−28.7 (3)
Ni1—N3—N4—C6	−174.8 (2)	N4—N3—Ni1—N5	143.9 (2)
C5—C6—N4—N3	0.5 (4)	C4—N3—Ni1—N5^i^	151.3 (3)
C1—N1—Ni1—N5	−33.6 (3)	N4—N3—Ni1—N5^i^	−36.1 (2)

Symmetry codes: (i) −*x*+1, −*y*+1, −*z*+2.

### Hydrogen-bond geometry (Å, °)

**Table:**

*D*—H···*A*	*D*—H	H···*A*	*D*···*A*	*D*—H···*A*
N4—H8···O1^ii^	0.88	1.94	2.797 (4)	164
N2—H4···O3	0.88	1.95	2.782 (3)	158

Symmetry codes: (ii) −*x*, −*y*+1, −*z*+2.
